# DUET: a graph-based workflow for TCR-epitope prioritization and tumor-reactive T-cell identification

**DOI:** 10.1093/bib/bbag519

**Published:** 2026-09-22

**Authors:** Yunsheng Chen, Vanessa Giuliano, Ian Dacillo, Wenjun Lin, Yan Yan, Ping Luo

**Affiliations:** Department of Computer Science & Mathematics, Faculty of Computer Science and Technology, Algoma University, 24 Queen St E, Brampton, ON L6V 1A3, Canada; Department of Computer Science & Mathematics, Faculty of Computer Science and Technology, Algoma University, 24 Queen St E, Brampton, ON L6V 1A3, Canada; Marshall McLuhan Catholic Secondary School, 1107 Avenue Rd, Toronto, ON M5N 3B1, Canada; Department of Computer Science & Mathematics, Faculty of Computer Science and Technology, Algoma University, 24 Queen St E, Brampton, ON L6V 1A3, Canada; College of Computational, Mathematical and Physical Sciences, School of Computer Science, University of Guelph, 474 Gordon St, Guelph, ON N1G 1Y4, Canada; Department of Computer Science & Mathematics, Faculty of Computer Science and Technology, Algoma University, 24 Queen St E, Brampton, ON L6V 1A3, Canada

**Keywords:** T-cell receptor, TCR-epitope binding, heterogeneous graph transformer, tumor-reactive T cells, single-cell RNA-seq

## Abstract

Accurate prioritization of T-cell receptor (TCR)-epitope interactions and identification of tumor-reactive T cells are important but difficult steps in immunotherapy-oriented bioinformatics workflows. Existing methods typically address these tasks separately and either model TCR-epitope pairs as independent observations or rely primarily on transcriptomic signatures. In this study, we present DUET (**D**ual **U**nified **E**valuation of **T**CR-Epitopes and Tumor-reactive T cells), a graph-based computational workflow that unifies both applications within a single heterogeneous graph framework. The protocol represents TCRs, epitopes, and T cells as typed nodes connected by similarity and association edges, and combines pretrained sequence embeddings with edge-aware graph attention, Laplacian positional encoding, and bidirectional cross-domain attention. Applied to the IEDB and VDJdb benchmarks, DUET achieved AUROC/AUPR values of 0.937/0.922 and 0.992/0.990, respectively, outperforming five state-of-the-art algorithms under standard evaluation. On a single-cell RNA-seq tumor-reactivity benchmark, the workflow achieved an area under the receiver operating characteristic curve of 0.985 and an area under the precision-recall curve of 0.975, substantially exceeding transcriptomic signature-based baselines. Additional generalization analyses showed that DUET’s clearest graph-specific benefit occurred under epitope-disjoint TCR-epitope prediction. Ablation analysis showed that Laplacian positional encoding provided the largest performance gain, particularly in sparse graph settings. These results suggest that heterogeneous graph modeling can serve as a practical protocol for integrating receptor sequence, antigen context, and cellular phenotype in computational immunology.

## Introduction

T-cell receptor (TCR) recognition of peptide antigens is central to adaptive immunity and underlies several immunotherapy strategies, including adoptive tumor-infiltrating lymphocyte transfer, TCR-engineered T-cell therapy, and neoantigen vaccine development [1]. However, in practice, two closely related computational problems remain difficult to solve at scale: prioritizing TCR-epitope pairs for downstream validation and identifying tumor-reactive T cells (TRT) from single-cell datasets without relying exclusively on labor-intensive functional assays. Public resources such as IEDB [2] and VDJdb [3] provide growing catalogs of validated TCR-epitope associations, while single-cell studies now jointly profile TCR sequence and cell state in tumor-infiltrating lymphocytes. What is still missing is a practical and reproducible workflow that can integrate these data types in a way that reflects the relational structure of immune repertoires rather than treating each observation in isolation.

Most existing computational approaches address only one part of this workflow. Earlier TCR specificity methods relied on sequence alignment, physicochemical scoring, or conventional machine learning over hand-crafted CDR3 features. Subsequent deep learning approaches, including ERGO-II [4], NetTCR-2.0 [5], DeepTCR [6], and PanPep [7], improved sequence representation learning but generally model each TCR-epitope pair as an independent prediction problem. This limits their ability to exploit repertoire-level structure, where similar TCRs may share binding preferences and related epitopes may recruit overlapping receptor sets. More recent graph-based methods such as GTE [8] and HeteroTCR [9] begin to address this issue for TCR-epitope prediction, but they remain focused on β-chain-centered binding tasks and do not extend naturally to TRT identification from single-cell data. Recent studies further highlight the importance of combining pretrained biomolecular representations with graph-based learning for interaction prediction. Graph-Regularized Attentive Protein Embeddings (GRAPE) applies graph-regularized protein language modeling to TCR-epitope binding prediction and addresses sparse interaction networks and class imbalance [10]. Beyond TCR-specific prediction, MGAPep integrates large language model embeddings with multimodal graph attention for protein-peptide binding-site prediction and cross-domain transfer [11], while MicroEnvPPI combines ESM-2-derived contextual embeddings, structural information, and graph contrastive learning to improve generalizable protein–protein interaction prediction [12]. These studies support the broader trend toward graph-enhanced pretrained representations for biomolecular interaction modeling, while Dual Unified Evaluation of TCR-Epitopes and Tumor-reactive T cells (DUET) extends this direction to a unified workflow covering both TCR-epitope prioritization and tumor-reactive T-cell identification. In parallel, current TRT workflows are dominated by transcriptomic signature scoring [13–16], with relatively limited use of TCR sequence context. As a result, users lack a single computational protocol that can connect receptor sequence, antigen association, and cellular phenotype in one analysis framework.

DUET is a graph-based computational workflow for two related use cases: TCR-epitope binding prioritization and tumor-reactive T-cell identification. The workflow represents TCRs, epitopes, and cells as nodes in a heterogeneous graph linked by intra-domain similarity edges and inter-domain association edges. Sequence-derived features are combined with edge-aware graph attention, Laplacian positional encoding (LPE) [17], and bidirectional cross-domain attention to propagate information within and across biological domains. This design enables the same core protocol to be applied to curated TCR-antigen databases and to single-cell datasets in which TCR clonotypes can be linked to transcriptional cell states. The workflow is evaluated on the IEDB dataset (8187 TCR-epitope pairs), the VDJdb dataset (71 686 pairs), and a gastrointestinal cancer single-cell dataset (376 functionally labeled T cells [18]). Ablation analysis is performed to determine which structural components contribute most strongly to predictive performance.

## Materials and methods

### Overview of the DUET workflow

As illustrated in Fig. 1, DUET is a graph-based computational workflow designed for two related applications: prioritizing TCR-epitope associations and identifying tumor-reactive T cells from single-cell RNA-seq data. For TCR-epitope prediction, the model takes paired TCR and epitope sequences as input, extracts modality-specific sequence representations, and constructs a heterogeneous TCR-epitope graph consisting of known associations together with TCR-TCR and epitope-epitope similarity edges. For TRT identification, DUET takes TCR sequences and scRNA-seq profiles as input and constructs a TCR-cell graph linking TCRs to their corresponding cells, while incorporating cell–cell similarity relationships derived from transcriptomic profiles. The resulting graph structures and node features are integrated through a structure injection module to enable information propagation across biologically related entities. DUET then applies modality-specific similarity attention mechanisms to refine TCR, epitope, and T-cell representations, followed by cross-attention layers that fuse information across modalities. The learned representations are subsequently used to predict either TCR-epitope associations or TRT states, depending on the application. In both settings, the workflow operates on a heterogeneous graph in which biologically distinct entities are represented as separate node types connected by similarity-based or association-based edges. The overall procedure consists of four stages, including data preparation, graph construction, feature encoding and graph-based representation learning, and task-specific prediction.

**Figure 1:**
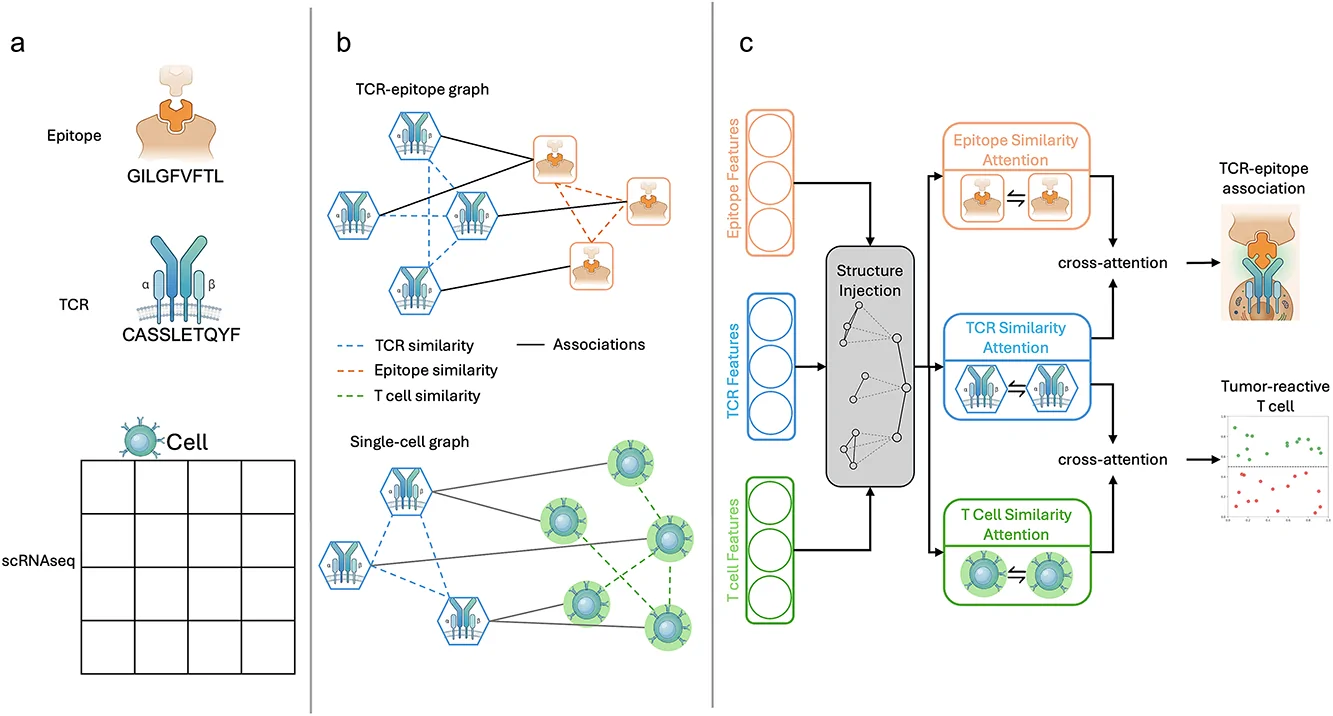
Overview of the DUET workflow. (a) Input modalities used by DUET. TCR-epitope prediction uses TCR and epitope sequences, whereas TRT identification uses TCR sequences and single-cell transcriptomic profiles. (b) Heterogeneous graph construction for the TCR-epitope task (top) and the single-cell task (bottom). Nodes represent TCRs, epitopes, or cells; dashed edges denote intra-domain similarity connections; solid edges denote inter-domain associations or clonotype membership. (c) Model architecture. Node features and structural encodings are injected via the structure injection module, followed by intra-domain graph attention within each node type and bidirectional cross-domain attention between domains. The resulting representations are used for TCR-epitope association prediction (edge-level) and tumor-reactive T-cell identification (node-level).

For TCR-epitope analysis, DUET takes curated TCR sequences and epitope sequences as input and returns a predicted binding score for each candidate TCR-epitope pair. For tumor-reactive T-cell analysis, DUET takes paired TCR and single-cell expression data as input and returns a predicted reactivity score for each cell. The same underlying workflow is used in both applications, with the main differences arising from the input node types and the prediction target.

### Benchmark datasets

Two public datasets were used to evaluate DUET for TCR-epitope prioritization. The IEDB dataset (version 3) comprised 8187 curated TCR-epitope pairs after sequence filtering, corresponding to 7682 unique TCRs and 980 unique epitopes. The VDJdb dataset (version 2025-12-29) comprised 71 686 curated pairs, representing 69 337 unique TCR α- and β-chain CDR3 sequences and 1352 unique epitopes.

For TRT identification, the gastrointestinal cancer dataset from Zheng et al. (Zheng2022) [18] was used. Following preprocessing and filtering for experimentally validated tumor-reactivity labels and complete paired α- and β-chain CDR3 sequences, the final dataset contained 376 cells, including 62 tumor-reactive and 314 non-reactive cells, representing 84 unique TCR clonotypes.

### Data preparation

For the IEDB and VDJdb benchmarks, only entries corresponding to *Homo sapiens* and major histocompatibility complex (MHC) class I were retained. Records were further filtered to include only those with complete curated α- and β-chain CDR3 sequences. Epitope sequences were restricted to canonical peptides composed of the 20 standard amino acids and ranging from 8 to 15 residues in length. TCR CDR3 sequences were further filtered to remove entries containing stop codons, and only sequences 8–20 residues in length were retained for both chains.

Raw count matrices from the Zheng2022 dataset were processed using Seurat v5 [19]. Quality-control filtering retained cells with 200–6000 detected genes and mitochondrial transcript fractions below 15%, followed by SCTransform normalization. To prevent information leakage, differential-expression feature selection was performed separately within each cross-validation fold. Specifically, differentially expressed genes were identified using only the training cells by a two-sided Wilcoxon rank-sum test, with a minimum detected-cell fraction of 0.10, an absolute log2 fold-change threshold of 0.25, and adjusted *P*-values below .05. The resulting fold-specific gene panel was then used to construct the expression features for both training and held-out cells.

For the TCR-epitope binding analyses, negative examples were generated by randomly sampling TCR-epitope pairs from the complete association space and excluding known positive associations. Negative samples were generated at a 1:1 ratio with positive associations. However, this global random-pair strategy does not preserve the TCR or epitope frequency distributions observed in the positive dataset. Moreover, because unobserved TCR-epitope pairs cannot be assumed to represent experimentally validated nonbinding interactions, three additional negative-sampling strategies were evaluated. First, positive-TCR-anchored random sampling retained the TCR from each positive pair and randomly assigned an unobserved epitope. Second, epitope-frequency-weighted sampling retained the positive TCR while sampling unobserved epitopes according to their frequency among known associations. Third, TCR-similarity-based hard-negative sampling paired each TCR with epitopes associated with one of its 20 nearest-neighbor TCRs but not observed for the query TCR. When no eligible hard negative could be identified, positive-TCR-anchored random sampling was used as a fallback strategy. These sensitivity analyses were performed to assess the robustness of model performance to different assumptions regarding negative-sample construction. For TRT prediction, we addressed class imbalance by using a class-weighted binary cross-entropy loss, assigning a higher loss weight to the minority tumor-reactive class based on the negative-to-positive sample ratio.

### Heterogeneous graph construction

DUET represents each dataset as a heterogeneous graph with typed nodes and edges. For the binding-prioritization task, the graph contains TCR nodes and epitope nodes. Inter-domain edges represent known TCR-epitope associations. Intra-domain edges are constructed separately among TCRs and among epitopes using k-nearest-neighbor (kNN) graphs derived from pairwise similarity.

TCR similarity is computed as a combination of k-mer-based cosine similarity and physicochemical similarity. Specifically, sequence-composition similarity was calculated from concatenated bigram (k = 2) and trigram (k = 3) frequency vectors, whereas physicochemical similarity was derived from five amino acid property scales from Aaindex [20], including hydrophobicity, normalized net charge, polarity, molecular weight, and isoelectric point. Pairwise cosine similarity was computed for both representations, and the resulting matrices were standardized before integration. For paired TCRs, α- and β-chain similarity matrices were combined as follows:


$$ {S}_{TCR}=\left(1-{w}_{\beta}\right){S}_{\alpha }+{w}_{\beta }{S}_{\beta } $$

where ${w}_{\beta }=0.7$ to reflect the greater contribution of the β chain to antigen specificity. The final TCR similarity matrix was then obtained by averaging the standardized k-mer-based and physicochemical similarity matrices. Epitope similarity is computed analogously, without chain weighting.

For the single-cell task, the graph contains TCR nodes and cell nodes. A cell is connected to the TCR clonotype it expresses through a membership edge. Additional kNN edges are constructed among TCRs based on sequence similarity and among cells based on expression similarity. This graph design allows DUET to capture both direct biological associations and neighborhood structure within each modality.

### Node feature initialization

DUET initializes each node type with domain-specific features before graph-based learning. TCR sequences are encoded using BertTCR [21], which is a BERT-based protein language model that generates contextualized embeddings for CDR3 sequences; α- and β-chain CDR3 embeddings are generated separately and concatenated to produce the TCR feature vector. Epitope sequences are encoded using the ESM-2 protein language model [22]. ESM-2 is a transformer-based masked language model trained on millions of protein sequences to capture deep evolutionary, structural, and functional representations of amino acids. Cell nodes are initialized using expression values for the differentially expressed genes selected exclusively from the corresponding training fold.

Before message passing, node features are augmented with structural information. Specifically, DUET incorporates LPE to capture graph-topological context, and similarity edge attributes to weight neighborhood aggregation by pairwise sequence similarity. The augmented node representation is defined as follows:


$$ {h}_i^{(0)}={x}_i+\frac{1}{\mid \mathcal{N}(i)\mid}\sum_{j\in \mathcal{N}(i)\mid } EdgeEnc\left({w}_{ij}\right)+ PE\left({\phi}_i\right) $$

where ${x}_i$ denotes the initial node feature, ${w}_{ij}$ is the edge weight between nodes *i* and its neighbor *j*, $EdgeEnc\left(\cdotp \right)$ maps the scalar edge weight to an additive bias term, and $PE\left({\phi}_i\right)$ is the projected LPE.

### Graph-based representation learning

The core of DUET consists of two complementary operations: intra-domain refinement and inter-domain information exchange.

#### Intra-domain refinement

Within each node type, DUET applies edge-aware graph transformer layers over the similarity graph. This step updates each node representation by aggregating information from its nearest neighbors while also incorporating edge weights directly into the attention mechanism. In practice, this allows more similar TCRs, epitopes, or cells to exert greater influence during representation learning.

For attention head *h*, the edge-aware attention logit between nodes *i*and *j* is computed as follows:


$$ {e}_{ij}^h=\frac{q_i^h\cdotp{k}_j^h}{\sqrt{d_h}}+{EdgeEnc}^h\left({w}_{ij}\right) $$

where *q* and *k* are learned query and key projections, ${d}_h$ is the per-head hidden dimension. Softmax normalization is then applied across the neighborhood of node *i*.

#### Inter-domain information exchange

After intra-domain refinement, DUET applies bidirectional cross-attention across the relevant biological domains. In the TCR-epitope workflow, this step allows TCR representations to attend to connected epitopes and vice versa. In the single-cell workflow, it allows information exchange between TCR clonotypes and their associated cells. This design enables the model to integrate sequence-level and cell-state information in a shared representation space.

### Task-specific prediction

DUET uses the learned graph representations in a task-specific prediction layer. For TCR-epitope prioritization, the final TCR and epitope embeddings for each candidate pair are concatenated and passed to a multilayer perceptron to generate a binding probability. For tumor-reactive T-cell identification, the final cell embeddings are passed to a multilayer perceptron to generate a per-cell tumor-reactivity score. Thus, the same workflow supports both edge-level prediction in the TCR-epitope setting and node-level prediction in the single-cell setting.

### Model configuration

DUET was implemented in PyTorch [23] and PyTorch Geometric [24], with pretrained sequence embeddings generated using Hugging Face model implementations. The same overall architecture was used across all three datasets, with task-specific input dimensions, prediction heads, and training hyperparameters adjusted for the TCR-epitope and single-cell workflows. The final hyperparameter settings and search ranges are provided in Supplementary Table S1. Detailed timing, throughput, inference latency, and memory measurements are also provided in Supplementary Tables S2 and S3.

### Model evaluation

DUET was evaluated using five repeated runs of 10-fold cross-validation across the three benchmark datasets. Predictive performance was assessed using the area under the receiver operating characteristic (ROC) curve (AUROC) and the area under the precision-recall (PR) curve (AUPR). The ROC and PR curves were plotted as mean curves across the repeated cross-validation runs, with shaded regions indicating the 95% confidence interval (CI) estimated from run-to-run variability.

For the TCR-epitope prediction, in each fold, known positive associations were partitioned into training (90%) and validation (10%) subsets, and an equal number of negative edges were sampled for each split. To prevent data leakage, inter-domain association edges used during message passing were restricted to positive edges from the training folds only. In contrast, validation-fold edges were excluded from the graph and used solely for held-out evaluation. For the single-cell analysis, differential-expression feature selection was nested within cross-validation. In each fold, gene selection was performed using only the training-cell labels. The cell feature matrix, cell–cell similarity graph, and positional encodings derived from these features were subsequently reconstructed for that fold. Held-out labels were not used during feature selection or graph construction. BertTCR embeddings and the TCR similarity graph were retained because they were derived solely from TCR sequences and were independent of tumor-reactivity labels.

For TCR-epitope prediction, DUET was compared with five state-of-the-art deep learning methods: DeepTCR, NetTCR-2.0, HeteroTCR, GTE, and PanPep. For tumor-reactive T-cell prediction, DUET was compared with four gene signature-based approaches (Hanada [14], Lowery [13], Meng [16], and Yossef [15]). Meng et al. reported two sets of signatures (tumor-reactive and non-tumor-reactive), so both variants were evaluated separately as Meng_TR and Meng_NTR. To ensure a fair comparison, all competing methods were trained and evaluated using identical cross-validation data splits. For each model, the TCR α-chain, β-chain, and epitope sequences were represented according to the encoding strategy specified in the original method. Since the majority of competing approaches were developed and benchmarked using the IEDB and VDJdb datasets, hyperparameters were configured following the settings recommended in the corresponding publications or official code repositories.

### Stringent generalization evaluation

To assess model generalization to previously unseen TCRs, epitopes, and patients, we performed additional stringent validation analyses. For the TCR-epitope binding task, we evaluated TCR-disjoint, epitope-disjoint, and both-disjoint validation schemes. In the TCR-disjoint setting, all test pairs contained TCRs absent from the training set. In the epitope-disjoint setting, all test pairs contained epitopes not observed during training. In the both-disjoint setting, neither the TCR nor the epitope in any test pair was present in the training data. These evaluation schemes assess the DUET’s ability to generalize beyond previously observed receptor and antigen sequences.

For the TRT prediction task, we additionally evaluated clonotype-disjoint and patient-disjoint validation. Patient-disjoint performance was reported as the macro AUROC across held-out donors containing both reactive and non-reactive cells, as AUROC is undefined for donors represented by a single class. One donor contained no reactive cells and was therefore excluded from this analysis.

Negative-sampling sensitivity was evaluated on both the IEDB and VDJdb datasets. In all three alternative sampling strategies, the TCR identities from the positive associations were retained, and only the selection of the decoy epitope differed. The negative-to-positive sampling ratio, model architecture, cross-validation protocol, and training hyperparameters were kept identical across experiments to ensure a fair comparison of negative-sampling effects. As the best performing competing algorithm, NetTCR-2.0 was also evaluated using the same sampled negative pairs and cross-validation splits as DUET to distinguish general sensitivity to negative set construction from model-specific effects.

### Comparison with non-graph and nonparametric baseline

To assess whether DUET provides benefits beyond simple retrieval, propagation, and feature-based approaches, we implemented a diverse set of non-graph and nonparametric baseline models for both prediction tasks.

For TCR-epitope binding prediction, the evaluated baselines included: (i) TCR-similarity kNN retrieval using the same TCR similarity graph employed by DUET, (ii) CDR3 sequence-distance kNN using TCRdist [25], and (iii) CDR3 sequence-similarity kNN using BLOSUM62-weighted alignment [26]. We also evaluated epitope-frequency prediction based on epitope occurrence in the training set, label propagation on the constructed graph, low-rank matrix completion of the TCR-epitope association matrix, logistic regression on concatenated BertTCR and ESM-2 embeddings, and a no-graph multilayer perceptron (MLP) using the same pretrained embeddings as DUET but without LPE, graph message passing, or cross-attention.

For TRT classification, we evaluated: (i) clone-size ranking; (ii) kNN retrieval on the cell–cell similarity graph; (iii) label propagation on the cell graph; (iv) logistic regression on the selected gene-expression panel; and (v) a no-graph MLP operating on transcriptomic features alone, without graph-transformer message passing, cross-attention, or graph-derived positional encoding.

All baseline methods were evaluated using the same training, validation, and test splits as DUET whenever applicable. For TCR-epitope binding prediction, validation and test associations were removed from the training graph prior to model fitting to prevent information leakage. Negative examples were treated as supervised non-edge labels and were not incorporated as graph edges during message passing. Baseline predictions were generated independently within each cross-validation fold using only training labels and training associations. For Zheng2022, all baselines were evaluated under random, clonotype-disjoint, and patient-disjoint validation protocols, as applicable. Performance was summarized using fold-averaged AUROC and AUPR, consistent with the primary DUET evaluation framework.

## Results

Using the evaluation framework, DUET was next compared with existing baseline methods across the IEDB, VDJdb, and Zheng2022 benchmarks. Across all three datasets, DUET achieved the highest performance in both ROC and PR analyses, with narrow 95% CI across five repeated runs, indicating strong predictive accuracy and stable model behavior.

As shown in Fig. 2, DUET achieved an AUROC of 0.937 (95% CI, 0.931–0.942) and an AUPR of 0.922 (95% CI, 0.909–0.936) on the IEDB benchmark. Among the five baselines, NetTCR-2.0 showed the highest overall performance (AUROC = 0.870, AUPR = 0.892), followed by HeteroTCR (AUROC = 0.814, AUPR = 0.843), while GTE, PanPep, and DeepTCR showed lower overall performance. The near-zero AUPR observed for DeepTCR likely reflects a mismatch between its generative architecture and the link-prediction setting, consistent with known limitations of repertoire-based generative models on pairwise association tasks.

**Figure 2:**
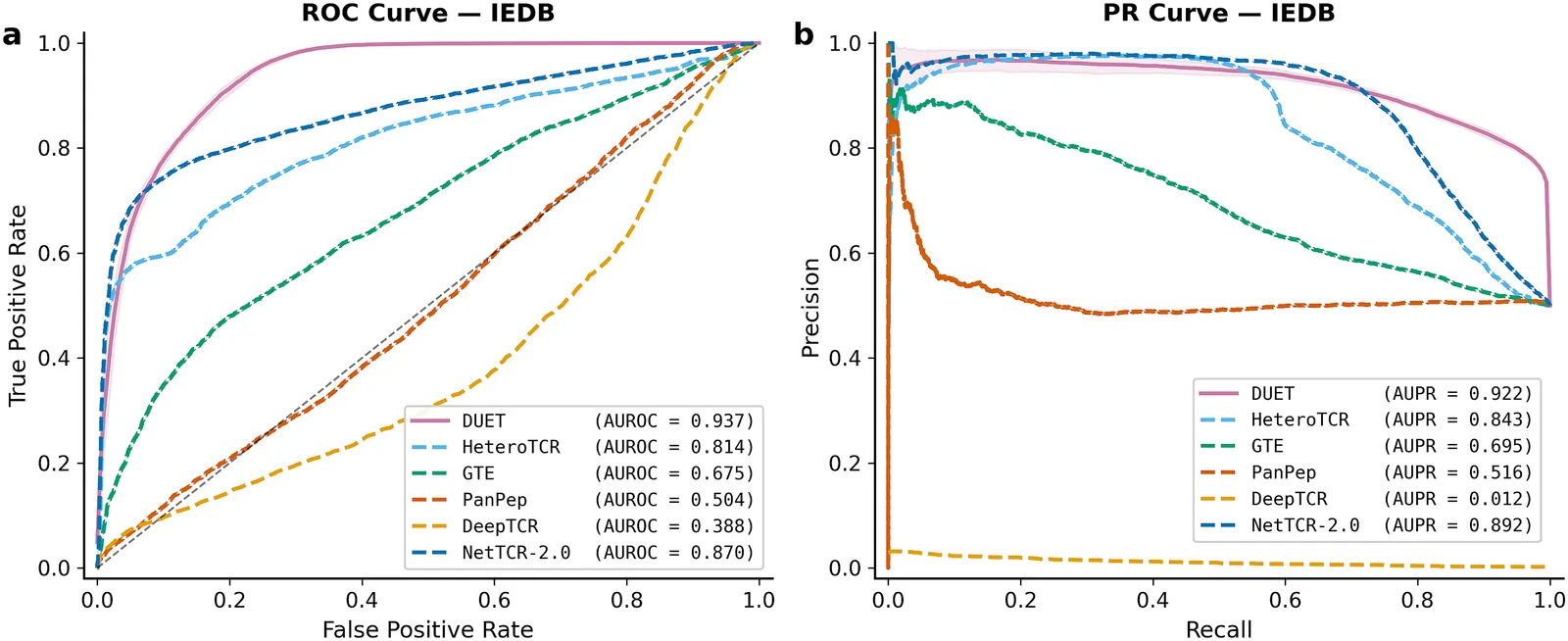
Predictive performance on the IEDB benchmark. (a) ROC curves and (b) PR curves for DUET and five baseline methods. AUROC and AUPR values are shown in the legend. Curves for DUET represent the mean across five repeated 10-fold cross-validation runs.

A similar trend was observed on the VDJdb benchmark (Fig. 3). DUET achieved an AUROC of 0.992 (95% CI, 0.991–0.993) and an AUPR of 0.990 (95% CI, 0.989–0.991), maintaining the top rank across both metrics. However, on this larger dataset, the top-performing methods converged to similarly high performance. While DUET outperformed competing approaches, the narrower performance margins relative to IEDB likely reflect the denser graph structure of VDJdb, which contains substantially more associations and provides a sufficient training signal for all graph-based methods to converge.

**Figure 3:**
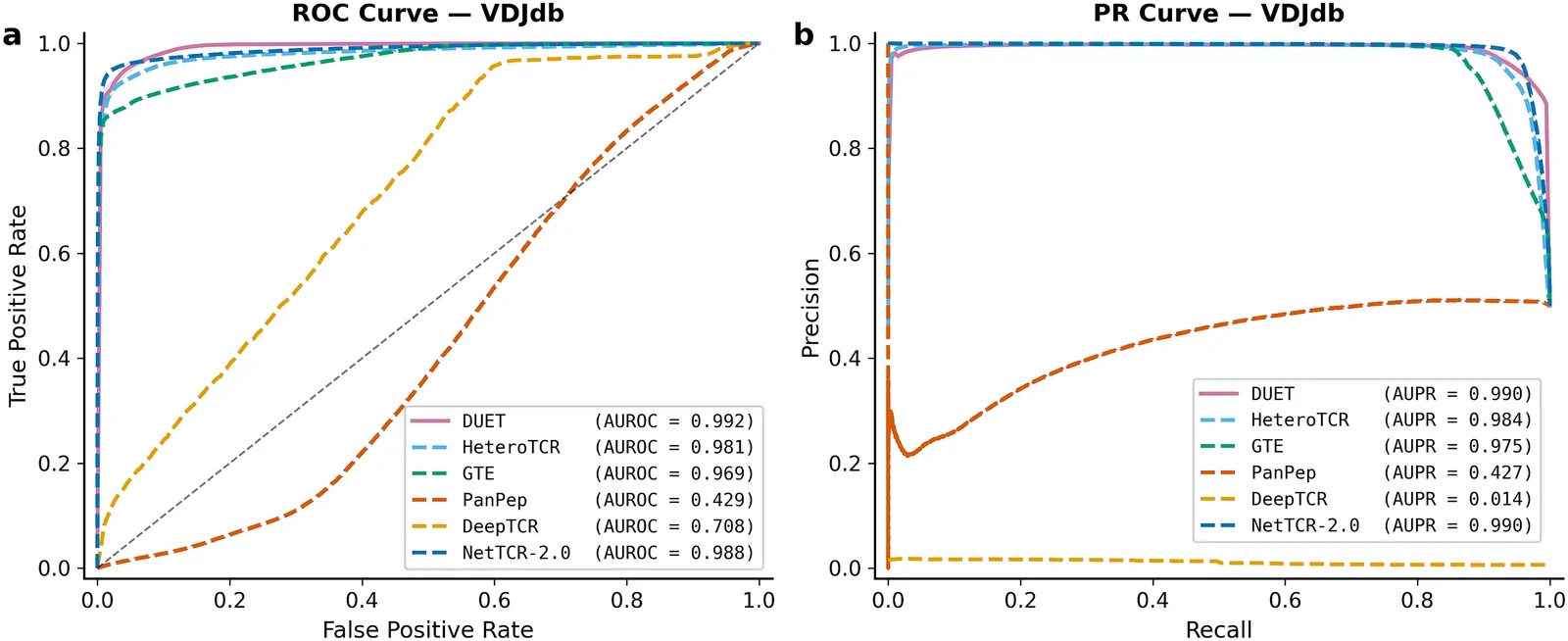
Predictive performance on the VDJdb benchmark. (a) ROC curves and (b) PR curves for DUET and five baseline methods.

On the Zheng2022 benchmark, DUET achieved an AUROC of 0.985 (95% CI, 0.981–0.990) and an AUPR of 0.975 (95% CI, 0.958–0.992), exceeding all evaluated signature-based baselines (Fig. 4). The best competing method, Lowery, obtained an AUROC of 0.735 and an AUPR of 0.310, whereas Hanada, Yossef, Meng_TR30, and Meng_NTR15 all showed substantially weaker discrimination. The AUPR gap is especially pronounced, where DUET’s AUPR of 0.975 represents a substantial improvement over the best baseline, indicating that signature-based approaches struggle to maintain precision when recovering the minority tumor-reactive class. These findings suggest that DUET captures tumor-reactivity signals more effectively by jointly modeling TCR sequence, clonotype-level graph structure, and transcriptional cell state, rather than relying on predefined gene expression programs.

**Figure 4:**
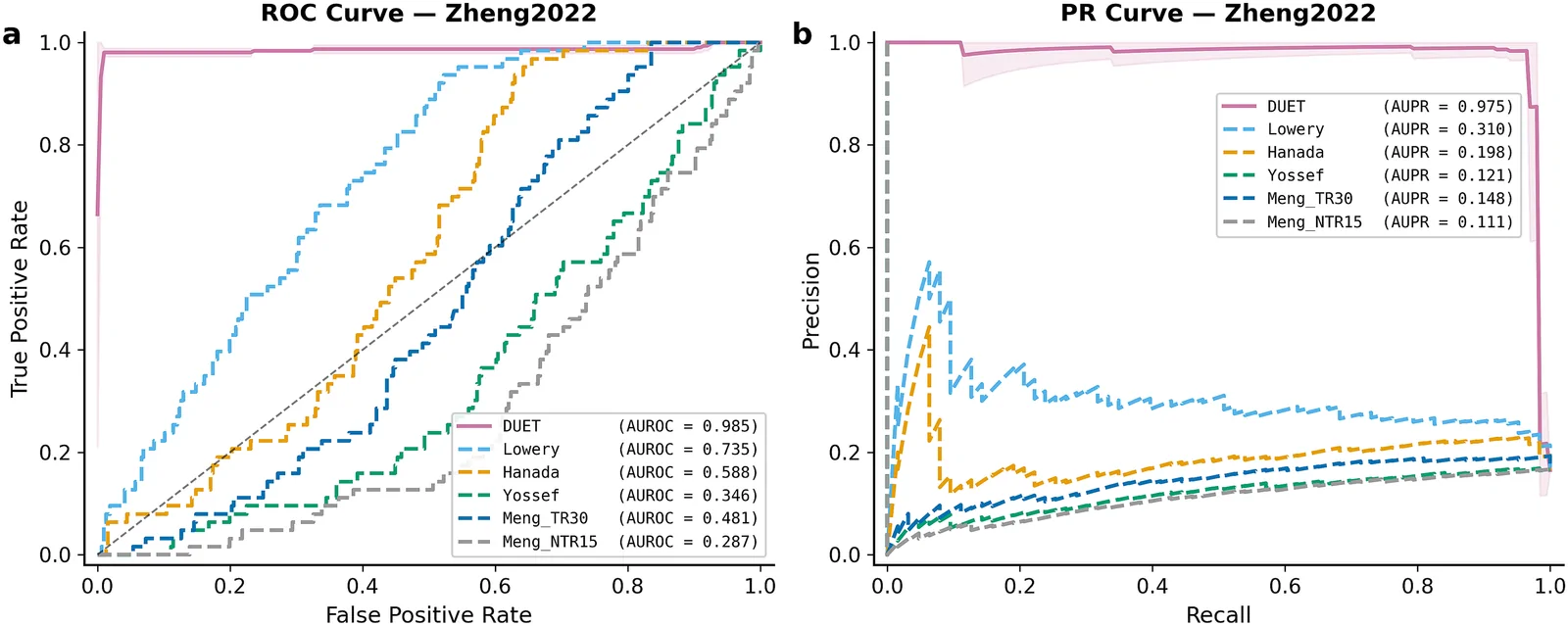
Predictive performance on the Zheng2022 single-cell benchmark. (a) ROC curves and (b) PR curves for DUET and five transcriptomic signature-based baselines.

### Sensitivity to negative-example selection

Negative-sampling sensitivity analysis demonstrated that model performance depended on the strategy used to construct presumed negative TCR-epitope pairs (Table 1). On IEDB, DUET consistently achieved higher performance than NetTCR-2.0 across all negative-sampling schemes, while both models exhibited substantial performance reductions under the more stringent frequency-weighted and TCR-similarity-based hard-negative settings. On VDJdb, both methods remained highly accurate across all sampling schemes, with only modest performance degradation under the harder negative constructions. Overall, DUET maintained competitive performance across all evaluation settings and showed robustness comparable to that of NetTCR-2.0 despite the increased difficulty of the alternative negative-sampling strategies. The results also indicate that random-pair negative sampling creates a comparatively easier prediction task, whereas frequency-weighted and TCR-similarity-based hard negatives provide a more stringent evaluation setting.

**Table 1:** Negative-sampling sensitivity analysis on IEDB and VDJdb.

Dataset	Model	Positive-TCR-anchored random sampling	Epitope-frequency-weighted sampling	TCR-similarity-based hard negative sampling
IEDB	DUET	0.894/0.906	0.707/0.711	0.761/0.758
IEDB	NetTCR-2.0	0.876/0.897	0.656/0.659	0.718/0.699
VDJdb	DUET	0.988/0.989	0.921/0.916	0.918/0.925
VDJdb	NetTCR-2.0	0.992/0.993	0.948/0.945	0.932/0.937

Note. Values are reported as AUROC/AUPR.

### Stringent generalization evaluation

To evaluate generalization beyond the standard random split, we added stringent generalization evaluation schemes (Table 2). For TCR-epitope binding, performance decreased as the held-out entities became more challenging. On IEDB, AUROC/AUPR decreased from 0.888/0.906 under TCR-disjoint validation to 0.756/0.672 under epitope-disjoint validation and 0.583/0.585 under both-disjoint validation. On VDJdb, the corresponding AUROC/AUPR values were 0.985/0.988, 0.895/0.798, and 0.728/0.696. These results indicate that random or TCR-disjoint splits are less stringent than unseen-epitope and both-disjoint evaluation, and that both-disjoint binding prediction remains challenging. For Zheng2022, DUET achieved AUROC/AUPR 0.996/0.991 under clonotype-disjoint validation and 0.902/0.760 under patient-disjoint validation, supporting evaluation beyond shared clonotypes while highlighting the need for larger patient-level cohorts.

**Table 2:** Performance under stringent generalization evaluation.

Dataset	Validation split	AUROC	AUPR
IEDB	TCR-disjoint	0.888	0.906
IEDB	Epitope-disjoint	0.756	0.672
IEDB	Both-disjoint	0.583	0.585
VDJdb	TCR-disjoint	0.985	0.988
VDJdb	Epitope-disjoint	0.895	0.798
VDJdb	Both-disjoint	0.728	0.696
Zheng2022	Clonotype-disjoint	0.996	0.991
Zheng2022	Patient-disjoint	0.902	0.760

### Graph-based learning improves generalization to unseen epitopes

To evaluate the contribution of graph-based representation learning, DUET was compared with a diverse set of baseline methods under identical validation protocols. These included retrieval-based approaches, propagation-based models, matrix-completion models, and feature-only models (Supplementary Table S4).

On standard random-split TCR-epitope benchmarks, several simple baselines achieved performance comparable to DUET, particularly on VDJdb. Logistic regression on pretrained embeddings and epitope-frequency scoring approached DUET performance, indicating that standard random splits contain substantial feature- and epitope-frequency-driven shortcuts. In contrast, under epitope-disjoint validation, DUET consistently achieved the highest AUROC and AUPR across both IEDB and VDJdb and substantially outperformed all non-graph and nonparametric baselines. These results suggest that graph-based representation learning contributes most strongly when generalizing to previously unseen epitopes, rather than merely exploiting sequence similarity or epitope frequency patterns.

Performance decreased for all methods under both-disjoint validation, where neither TCRs nor epitopes were observed during training, and the advantage of DUET over the no-graph MLP became considerably smaller (Table 3). This finding indicates that simultaneous generalization to unseen receptors and unseen epitopes remains challenging and is not fully addressed by graph propagation alone.

**Table 3:** Key diagnostic comparisons for graph-specific benefit.

Dataset	Validation setting	DUET	No-graph MLP	Best simple baseline (logistic regression)
IEDB	Epitope-disjoint	0.756/0.672	0.591/0.589	0.516/0.530
VDJdb	Epitope-disjoint	0.895/0.798	0.733/0.682	0.663/0.645
Zheng2022	Patient-disjoint	0.902/0.760	0.905/0.746	0.872/0.687

Note. For IEDB/VDJdb, logistic regression used BertTCR/ESM-2 embeddings, for Zheng2022, logistic regression used the selected gene panel. Full retrieval results are reported in Supplementary Table S4.

For Zheng2022 tumor-reactive T-cell classification, logistic regression and the no-graph MLP achieved performance comparable to DUET under patient-disjoint validation (Table 3), indicating that transcriptomic features explain much of the predictive signal in this cohort. Therefore, while DUET provides a unified framework for integrating transcriptomic, TCR, and graph-derived information, the strongest evidence for graph-based learning was observed in epitope-disjoint TCR-epitope prediction rather than tumor-reactive T-cell classification.

### Ablation study

To isolate the contribution of each structural encoding component, three progressively enriched model variants were evaluated across all three benchmarks. These included a base model with no structural encoding, the base model augmented with LPE, and the full model that further incorporates similarity edge attributes (Base + LPE + Edge Attributes). Results are shown in Fig. 5.

**Figure 5:**
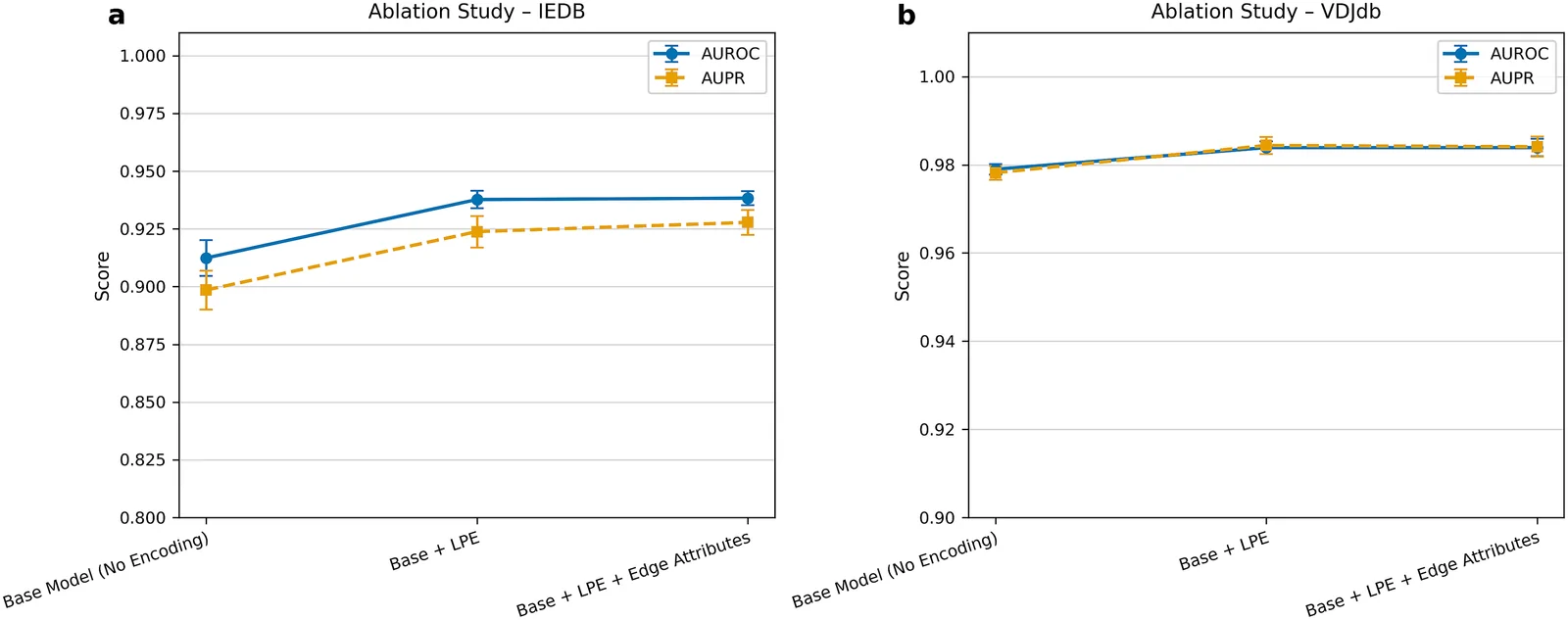
Ablation study results. Mean AUROC and AUPR scores (±95% CI) for three progressively enriched model variants on (a) IEDB and (b) VDJdb. The three variants are: base model (no structural encoding), Base + LPE, and Base + LPE + Edge Attributes.

On the IEDB benchmark (Fig. 5a), removing all structural encodings yielded a base performance of AUROC = 0.912 and AUPR = 0.899. Adding LPE produced a substantial and consistent improvement, raising AUROC to 0.938 and AUPR to 0.924. This finding indicates that graph-topological context captured by LPE is informative in this setting, likely because the IEDB graph is comparatively sparse and degree-skewed, whereas local spectral structure provides signals that are not recoverable from node features alone. The addition of similarity edge attributes yielded only a marginal increment (AUROC = 0.938, AUPR = 0.928), suggesting that edge weights contribute modestly once positional structure is encoded.

On the VDJdb benchmark (Fig. 5b), all three variants converged to near-ceiling performance. The base model already achieved an AUROC of 0.979 and AUPR of 0.978, and the addition of LPE and edge attributes produced incremental improvements that remained within the confidence interval range. This near-uniform performance across variants is consistent with the substantially larger and denser graph structure of VDJdb, which contains more TCR-epitope associations and provides sufficient training signal for all model variants to converge regardless of structural encoding.

Notably, degree encoding was also evaluated as an additional structural component. Contrary to expectations, incorporating degree encoding consistently degraded performance across both the IEDB and VDJdb benchmarks (Supplementary Figs S1 and S2). This can be attributed to the highly skewed degree distribution in the TCR-epitope graphs, where hubs may cause degree embeddings to introduce a confounding bias rather than a useful structural signal. Based on these results, degree encoding was excluded from the final DUET architecture. The ablation results also suggest that LPE provides the most consequential structural signal among the tested components, particularly in data-scarce or graph-sparse regimes such as IEDB where topological context is otherwise difficult to recover from node features and connectivity alone.

On the scRNA dataset, all variants achieved saturated performance under random-split evaluation (AUROC and AUPR of 1, Supplementary Fig. S3). As a result, we further evaluated DUET against non-graph baselines under progressively stricter validation settings (Fig. 6). Under random split, clone-size ranking, logistic regression on the selected gene panel, kNN on the cell–cell similarity graph, and label propagation all achieved non-trivial performance, confirming that the Zheng2022 cohort contains strong transcriptomic and clonotype-associated signals. However, DUET remained strongest under the clonotype-disjoint setting and retained competitive performance under patient-disjoint validation, where all methods showed wider uncertainty due to the small number of held-out donors and class imbalance. These results indicate that the scRNA benchmark is not well suited for distinguishing fine-grained architectural components under random splits, but the stricter non-graph baseline comparison supports DUET as a useful integrative framework while also showing that larger multi-patient TRT datasets will be needed to determine the graph-specific contribution more definitively.

**Figure 6:**
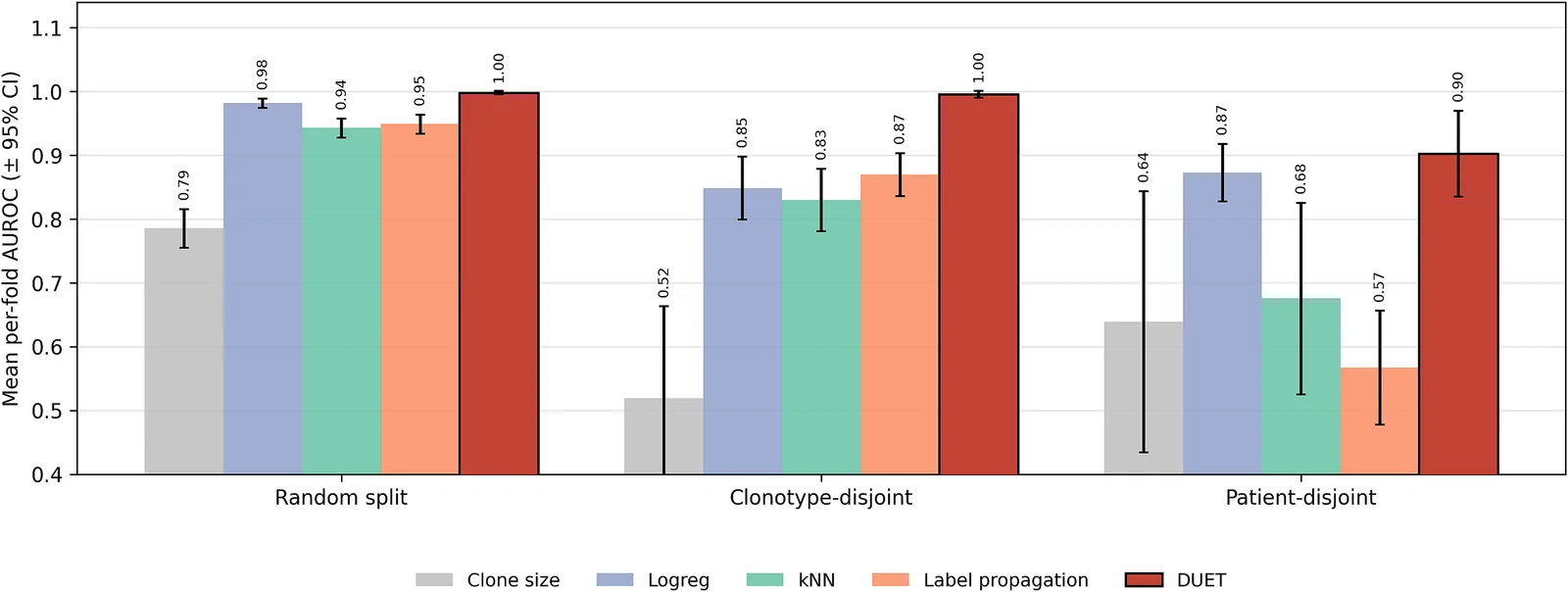
Non-graph baseline comparison for the Zheng2022 benchmark. Mean per-fold AUROC with 95% CI under random, clonotype-disjoint, and patient-disjoint validation. DUET performed strongest under clonotype-disjoint validation and remained competitive under patient-disjoint validation.

## Discussion

DUET showed strong predictive performance across both TCR-epitope prioritization and tumor-reactive T-cell identification, while the expanded validation analyses clarify where graph-based learning provides the clearest benefit. Under standard random and TCR-disjoint evaluation, DUET and simpler feature-based models both performed well, indicating that pretrained sequence embeddings, epitope frequency, and local similarity structure capture substantial predictive signal when related entities remain represented during training. In contrast, the most pronounced graph-specific advantage was observed under epitope-disjoint validation. In this setting, DUET consistently outperformed the no-graph MLP as well as retrieval, propagation, matrix-completion, and linear baselines on both IEDB and VDJdb. These results suggest that heterogeneous message passing is particularly useful when transferring information to epitopes without direct training associations, a setting that better reflects practical prioritization of less-characterized antigens.

The structural ablation results further support this interpretation. LPE produced the largest improvement on IEDB, where the association graph is sparser and more heterogeneous, suggesting that spectral topological information helps recover structure that is not fully captured by node features alone. Similarity edge attributes provided smaller additional gains, indicating that explicit edge-weight information can complement positional structure but was not the dominant driver of performance in the current benchmarks. On VDJdb, all structural variants approached near-ceiling performance, likely because the larger and denser graph provides sufficient training signal even with simpler structural encodings. Thus, the contribution of individual graph components appears to be dataset-dependent, with the strongest gains occurring in sparser or more difficult generalization settings.

For TRT identification, the Zheng2022 analysis provides a strong proof-of-concept for applying DUET to paired TCR and single-cell transcriptomic data. DUET achieved high performance under both clonotype-disjoint and patient-disjoint validation, indicating that the workflow can generalize beyond shared clonotypes and random cell-level splits. However, logistic regression on selected transcriptomic features and the no-graph MLP also performed strongly, especially under patient-disjoint validation. Therefore, these results support DUET as a practical integrative framework for TRT prediction, but they should not be interpreted as definitive evidence that graph propagation is required for this task. Larger multi-patient and cross-cohort single-cell datasets [27] will be needed to determine whether graph structure provides consistent additional value beyond transcriptomic cell-state and clonal-expansion signals.

Several limitations should be considered when interpreting these results. First, the TCR-epitope binding task relies on sampled unobserved pairs as presumed negatives because experimentally verified nonbinding pairs remain scarce. The negative-sampling sensitivity analysis showed that performance decreased under frequency-weighted and TCR-similarity hard-negative schemes, particularly on IEDB, confirming that random negative sampling can overestimate performance. Second, although disjoint validation provides a more stringent internal assessment than random cross-validation, it is not equivalent to fully independent external validation. Future work should evaluate DUET on prospectively generated TCR-epitope datasets and independent single-cell tumor-reactivity cohorts.

An additional limitation is that DUET does not explicitly model MHC/HLA context. TCR recognition occurs in the context of peptide–MHC complexes, and peptide-only epitope representations cannot fully capture HLA-dependent presentation and binding effects. Future versions of DUET could incorporate HLA alleles as additional graph nodes, integrate learned HLA embeddings, or model full peptide–MHC features directly. Such extensions would be particularly important for neoantigen prioritization and personalized immunotherapy applications.

In summary, DUET provides a unified heterogeneous graph workflow for integrating receptor sequence, antigen context, and single-cell phenotype. Our analyses support a focused conclusion: DUET performs strongly across the evaluated tasks, with its clearest graph-specific benefit observed in unseen-epitope TCR-epitope prioritization, while the Zheng2022 results demonstrate promising proof-of-concept performance for TRT identification. This framework provides a practical foundation for future HLA-aware and externally validated models for T-cell repertoire and immunotherapy-oriented prediction tasks.

## Conclusion

DUET provides a graph-based computational workflow for two related immunological tasks, namely prioritizing TCR-epitope associations and identifying tumor-reactive T cells from single-cell data. By integrating sequence-derived representations with similarity-aware graph structure and cross-domain attention, the protocol captures both molecular and repertoire-level context in a unified framework. Across the IEDB, VDJdb, and Zheng2022 benchmarks, DUET showed strong predictive performance, with particularly favorable precision-recall behavior relative to competing approaches. Ablation analysis further indicated that LPE is the most consequential structural component, particularly in sparse and degree-skewed graph settings where topological context is otherwise difficult to recover from node features alone. Taken together, these results support heterogeneous graph modeling as a practical strategy for linking TCR sequence, antigen specificity, and cellular phenotype, positioning DUET as a useful protocol for bioinformatics workflows in immunotherapy and T-cell repertoire analysis.

**Key Points**

- DUET is a heterogeneous graph-based workflow that unifies TCR-epitope binding prioritization and tumor-reactive T cell identification within a single computational framework.
- On the IEDB and VDJdb benchmarks, DUET outperformed five state-of-the-art methods, achieving AUROC/AUPR of 0.937/0.922 and 0.992/0.990, respectively.
- On a gastrointestinal cancer single-cell dataset, DUET achieved strong tumor-reactive T-cell prediction performance under random cross-validation and remained competitive under clonotype- and patient-disjoint validation.
- Ablation analysis demonstrated that Laplacian positional encoding contributes most meaningfully in sparse, degree-skewed graph settings, while all variants converged under denser graph conditions.
- Stringent generalization validation showed that DUET’s clearest graph-specific benefit occurs under epitope-disjoint TCR-epitope prediction, while both-disjoint binding and patient-disjoint TRT prediction remain challenging.

## Supplementary Material

Web_Material_bbag519

## Data Availability

Our code and data are publicly available at: https://github.com/GenTensor/DUET.
