# Supplementary material for: DUET: a graph-based workflow for TCR-epitope prioritization and tumor-reactive T-cell identification
Source: Brief Bioinform. 2026 Sep 22;27(5):bbag519. doi: 10.1093/bib/bbag519 (PMC13631928; doi:10.1093/bib/bbag519)
Supplement: Supplementary file 1 [file Web_Material_bbag519.docx]

Supplementary Material

# Supplementary Methods

Hyperparameter tuning was performed on the IEDB benchmark using a grid search over the candidate values listed in Table S1. Tuned hyperparameters included the number of graph transformer layers, hidden-channel dimensionality, the number of attention heads, and the number of nearest neighbors (K) used for graph construction. Other hyperparameters, including the dropout rate, weight decay, learning rate, and Laplacian positional-encoding dimensionality, were chosen based on experience.

The hyperparameter combination achieving the highest mean AUROC on IEDB was selected as the primary architectural configuration. This configuration was used for the binding benchmarks, with task-specific input dimensions and prediction heads adjusted as required for VDJdb and Zheng2022. This strategy reduced dataset-specific hyperparameter optimization while preserving necessary task-specific adaptations.

# Supplementary Table S1

| Hyperparameter | Ranges | Selected value |
| --- | --- | --- |
| No. of transformer layers | 1, 2, 3 | 2 |
| Hidden channel dimensionality | 64, 128, 256 | 256 |
| No. of attention heads | 2, 4, 8 | 2 |
| No. of nearest neighbors | 10, 15, 20, 25 | 20 (TCR), 15 (Epitope) |
| Dropout | N/A | 0.3 |
| Weight decay | N/A | 1e-4 |
| Learning rate | N/A | 5e-4 |
| Dimensionality of LPE encoding | N/A | 8 |

# Supplementary Table S2

Pretrained encoder runtime and memory usage

| Dataset | Encoder | Model load time | Encoding time | Peak encoding RAM |
| --- | --- | --- | --- | --- |
| IEDB | BertTCR | 0.4 s | 1 min 49 s | 0.897 GB |
| IEDB | ESM-2 | 2.7 s | 47.5 s | 3.699 GB |
| VDJdb | BertTCR | 0.3 s | 15 min 14 s | 1.642 GB |
| VDJdb | ESM-2 | 2.7 s | 1 min 04 s | 3.735 GB |
| Zheng2022 | BertTCR | 0.4 s | 1.1 s | 1.575 GB |

# Supplementary Table S3

Graph-transformer runtime and GPU memory usage

| Dataset | Graph construction time | Training time / fold | Peak training GPU memory | Inference time / fold | Peak inference GPU memory |
| --- | --- | --- | --- | --- | --- |
| IEDB | 10.7 s | 5.3 ± 0.1 s | 3.732 GB | 11.6 ms | 1.350 GB |
| VDJdb | 35.4 s | 46.9 s | 14.682 GB | 44.7 ms | 5.424 GB |
| Zheng2022 | 1.6 s | 1.2 ± 0.3 s | 0.602 GB | 4.8 ms | 0.557 GB |

# Supplementary Table S4

Complete diagnostic comparisons for graph-specific benefit. Values are macro fold-averaged AUROC/AUPR.

| Dataset | Validation split | Method | AUROC / AUPR |
| --- | --- | --- | --- |
| IEDB | Random | DUET | 0.937 / 0.921 |
| IEDB | Random | TCR-similarity kNN (DUET graph) | 0.799 / 0.802 |
| IEDB | Random | Epitope-frequency baseline | 0.864 / 0.895 |
| IEDB | Random | Label propagation | 0.656 / 0.658 |
| IEDB | Random | Matrix completion | 0.519 / 0.642 |
| IEDB | Random | Logistic regression on embeddings | 0.869 / 0.886 |
| IEDB | Random | TCRdist kNN | 0.805 / 0.751 |
| IEDB | Random | BLOSUM62 kNN | 0.823 / 0.862 |
| IEDB | Random | No-graph MLP | 0.886 / 0.905 |
| IEDB | TCR-disjoint | DUET | 0.887 / 0.906 |
| IEDB | TCR-disjoint | TCR-similarity kNN (DUET graph) | 0.800 / 0.802 |
| IEDB | TCR-disjoint | Epitope-frequency baseline | 0.865 / 0.896 |
| IEDB | TCR-disjoint | Label propagation | 0.646 / 0.646 |
| IEDB | TCR-disjoint | Matrix completion | 0.500 / 0.500 |
| IEDB | TCR-disjoint | Logistic regression on embeddings | 0.862 / 0.883 |
| IEDB | TCR-disjoint | TCRdist kNN | 0.807 / 0.754 |
| IEDB | TCR-disjoint | BLOSUM62 kNN | 0.824 / 0.864 |
| IEDB | TCR-disjoint | No-graph MLP | 0.887 / 0.907 |
| IEDB | Epitope-disjoint | DUET | 0.756 / 0.672 |
| IEDB | Epitope-disjoint | TCR-similarity kNN (DUET graph) | 0.500 / 0.500 |
| IEDB | Epitope-disjoint | Epitope-frequency baseline | 0.500 / 0.500 |
| IEDB | Epitope-disjoint | Label propagation | 0.516 / 0.519 |
| IEDB | Epitope-disjoint | Matrix completion | 0.499 / 0.503 |
| IEDB | Epitope-disjoint | Logistic regression on embeddings | 0.516 / 0.529 |
| IEDB | Epitope-disjoint | TCRdist kNN | 0.500 / 0.500 |
| IEDB | Epitope-disjoint | BLOSUM62 kNN | 0.500 / 0.500 |
| IEDB | Epitope-disjoint | No-graph MLP | 0.591 / 0.589 |
| IEDB | Both-disjoint | DUET | 0.583 / 0.585 |
| IEDB | Both-disjoint | TCR-similarity kNN (DUET graph) | 0.500 / 0.500 |
| IEDB | Both-disjoint | Epitope-frequency baseline | 0.500 / 0.500 |
| IEDB | Both-disjoint | Label propagation | 0.500 / 0.500 |
| IEDB | Both-disjoint | Matrix completion | 0.500 / 0.500 |
| IEDB | Both-disjoint | Logistic regression on embeddings | 0.531 / 0.542 |
| IEDB | Both-disjoint | TCRdist kNN | 0.500 / 0.500 |
| IEDB | Both-disjoint | BLOSUM62 kNN | 0.500 / 0.500 |
| IEDB | Both-disjoint | No-graph MLP | 0.591 / 0.619 |
| VDJdb | Random | DUET | 0.992 / 0.991 |
| VDJdb | Random | TCR-similarity kNN (DUET graph) | 0.953 / 0.953 |
| VDJdb | Random | Epitope-frequency baseline | 0.978 / 0.984 |
| VDJdb | Random | Label propagation | 0.917 / 0.917 |
| VDJdb | Random | Matrix completion | 0.471 / 0.619 |
| VDJdb | Random | Logistic regression on embeddings | 0.990 / 0.989 |
| VDJdb | Random | TCRdist kNN | 0.918 / 0.822 |
| VDJdb | Random | BLOSUM62 kNN | 0.960 / 0.968 |
| VDJdb | Random | No-graph MLP | 0.991 / 0.991 |
| VDJdb | TCR-disjoint | DUET | 0.985 / 0.988 |
| VDJdb | TCR-disjoint | TCR-similarity kNN (DUET graph) | 0.953 / 0.953 |
| VDJdb | TCR-disjoint | Epitope-frequency baseline | 0.978 / 0.984 |
| VDJdb | TCR-disjoint | Label propagation | 0.919 / 0.917 |
| VDJdb | TCR-disjoint | Matrix completion | 0.500 / 0.500 |
| VDJdb | TCR-disjoint | Logistic regression on embeddings | 0.987 / 0.987 |
| VDJdb | TCR-disjoint | TCRdist kNN | 0.917 / 0.826 |
| VDJdb | TCR-disjoint | BLOSUM62 kNN | 0.960 / 0.968 |
| VDJdb | TCR-disjoint | No-graph MLP | 0.988 / 0.989 |
| VDJdb | Epitope-disjoint | DUET | 0.895 / 0.798 |
| VDJdb | Epitope-disjoint | TCR-similarity kNN (DUET graph) | 0.500 / 0.500 |
| VDJdb | Epitope-disjoint | Epitope-frequency baseline | 0.500 / 0.500 |
| VDJdb | Epitope-disjoint | Label propagation | 0.518 / 0.526 |
| VDJdb | Epitope-disjoint | Matrix completion | 0.502 / 0.504 |
| VDJdb | Epitope-disjoint | Logistic regression on embeddings | 0.663 / 0.644 |
| VDJdb | Epitope-disjoint | TCRdist kNN | 0.500 / 0.500 |
| VDJdb | Epitope-disjoint | BLOSUM62 kNN | 0.500 / 0.500 |
| VDJdb | Epitope-disjoint | No-graph MLP | 0.733 / 0.682 |
| VDJdb | Both-disjoint | DUET | 0.728 / 0.696 |
| VDJdb | Both-disjoint | TCR-similarity kNN (DUET graph) | 0.500 / 0.500 |
| VDJdb | Both-disjoint | Epitope-frequency baseline | 0.500 / 0.500 |
| VDJdb | Both-disjoint | Label propagation | 0.500 / 0.500 |
| VDJdb | Both-disjoint | Matrix completion | 0.500 / 0.500 |
| VDJdb | Both-disjoint | Logistic regression on embeddings | 0.656 / 0.643 |
| VDJdb | Both-disjoint | TCRdist kNN | 0.500 / 0.500 |
| VDJdb | Both-disjoint | BLOSUM62 kNN | 0.500 / 0.500 |
| VDJdb | Both-disjoint | No-graph MLP | 0.715 / 0.655 |
| Zheng2022 | Random | DUET | 0.985 / 0.975 |
| Zheng2022 | Random | Clone-size ranking | 0.785 / 0.483 |
| Zheng2022 | Random | Cell-graph kNN | 0.943 / 0.875 |
| Zheng2022 | Random | Label propagation | 0.949 / 0.893 |
| Zheng2022 | Random | Logistic regression on gene panel | 0.982 / 0.948 |
| Zheng2022 | Clonotype-disjoint | DUET | 0.996 / 0.991 |
| Zheng2022 | Clonotype-disjoint | Clone-size ranking | 0.519 / 0.485 |
| Zheng2022 | Clonotype-disjoint | Cell-graph kNN | 0.830 / 0.496 |
| Zheng2022 | Clonotype-disjoint | Label propagation | 0.870 / 0.656 |
| Zheng2022 | Clonotype-disjoint | Logistic regression on gene panel | 0.848 / 0.670 |
| Zheng2022 | Clonotype-disjoint | No-graph MLP | 0.979 / 0.958 |
| Zheng2022 | Patient-disjoint | DUET | 0.902 / 0.760 |
| Zheng2022 | Patient-disjoint | Clone-size ranking | 0.638 / 0.425 |
| Zheng2022 | Patient-disjoint | Cell-graph kNN | 0.675 / 0.334 |
| Zheng2022 | Patient-disjoint | Label propagation | 0.567 / 0.221 |
| Zheng2022 | Patient-disjoint | Logistic regression on gene panel | 0.872 / 0.687 |
| Zheng2022 | Patient-disjoint | No-graph MLP | 0.904 / 0.746 |

# Supplementary Figures

Figure S1. Ablation Study with Degree Encoding (IEDB)

Figure S2. Ablation Study with Degree Encoding (VDJdb)

Figure S3. Ablation Study with Degree Encoding (Zheng2022)
